# Intensive Safety Monitoring of Rituximab (Biosimilar Novex^®^ and the Innovator) in Pediatric Patients With Complex Diseases

**DOI:** 10.3389/fphar.2021.785770

**Published:** 2022-01-26

**Authors:** Natalia Riva, Manuel Molina, Berta L. Cornaló, María V. Salvador, Andrea Savransky, Silvia Tenembaum, María M. Katsicas, Marta Monteverde, Paulo Cáceres Guido, Marcela Rousseau, Raquel Staciuk, Agustín González Correas, Pedro Zubizarreta, Oscar Imventarza, Eduardo Lagomarsino, Eduardo Spitzer, Marcelo Tinelli, Paula Schaiquevich

**Affiliations:** ^1^ Unit of Innovative Treatments, Hospital de Pediatría JP Garrahan, Buenos Aires, Argentina; ^2^ Consejo Nacional de Investigaciones Científicas y Técnicas, CONICET, Buenos Aires, Argentina; ^3^ Pharmacy, Hospital de Pediatría JP Garrahan, Buenos Aires, Argentina; ^4^ Neurology Service, Hospital de Pediatría JP Garrahan, Buenos Aires, Argentina; ^5^ Immunology and Rheumatology Service, Hospital de Pediatría JP Garrahan, Buenos Aires, Argentina; ^6^ Nephrology Unit, Hospital de Pediatría JP Garrahan, Buenos Aires, Argentina; ^7^ Unit of Clinical Pharmacokinetics, Pharmacy, Hospital de Pediatría JP Garrahan, Buenos Aires, Argentina; ^8^ Health Technology Assessment Coordination, Hospital de Pediatría JP Garrahan, Buenos Aires, Argentina; ^9^ Bone Marrow Transplant Service, Hospital de Pediatría JP Garrahan, Buenos Aires, Argentina; ^10^ Hematology and Oncology Service, Hospital de Pediatría JP Garrahan, Buenos Aires, Argentina; ^11^ Liver Transplant Service, Hospital de Pediatría JP Garrahan, Buenos Aires, Argentina; ^12^ Laboratorio Elea-Phoenix S.A., Scientific Department, Los Polvorines, Argentina

**Keywords:** Rituximab, Biosimilar Pharmaceuticals, Monoclonal Antibody, Pediatric, Adverse Drug Reactions, Hypersensitivity, Risk Factors

## Abstract

Although rituximab is widely used off-label for complex pediatric diseases, safety reports are limited. We aimed to report evidence of its use in clinical practice, to describe the incidence of adverse drug reactions (ADR) to rituximab biosimilar Novex^®^ and innovator, and to identify risk factors for the development of ADR in a real-life follow-up cohort of pediatric patients with complex diseases. We conducted a prospective, longitudinal, observational, single-centre study in patients that received rituximab for any complex disease, and as part of an intensive pharmacovigilance program. Demographic, pharmacological, clinical, and drug-related data were collected for all patients. ADR-free survival, including infusion-related reactions (IRR) and delayed ADR (dADR), was estimated using Kaplan-Meier curves. Risk factors were evaluated by multivariable Cox regression models. In total, 77 patients (<19 y.o.) received 187 infusions of rituximab Novex^®^ (*n* = 155) or innovator rituximab (*n* = 32) for neurologic (Neu), immune-hematologic-rheumatic (IHR), oncologic (O) diseases, and hematopoietic stem-cell transplantation (HSCT) or solid-organ transplantation (SOT). We recorded 29 IRR and 58 dADR that occurred in 27 (35.1%) and 29 (37.7%) patients, respectively. The respiratory tract was the most affected during IRR (29.6%) and hypogammaglobulinemia (37.9 %) was the most frequent dADR. First *versus* subsequent infusions (HR 5.4, CI95% 2.4–12.1, *p*<0.05), sex (boys *vs.* girls, HR 0.3, CI95% 0.1–0.8, and *p*<0.05), and diagnosis (Neu-IHR diseases *vs.* O-HSCT-SOT, HR 2.3, CI95% 1.02–5.4, and *p* < 0.05) were significantly associated with the development of IRR. For dADR, risk factors were diagnosis (Neu-IHR diseases *vs.* O-HSCT-SOT, HR 0.4, CI95% 0.2–0.9, and *p* < 0.05) and cumulative body surface area-normalized dosage (HR 1.0003, CI95% 1.0001–1.0006, and *p* < 0.05). The present is the largest real-world safety assessment of rituximab in Latin-American children with complex diseases supporting its use based on the overall acceptable safety. Identification of risk factors may contribute to optimization of off-label rituximab treatment in pediatrics.

## Introduction

Biological drugs are the basis for targeted therapy, improving therapeutic efficacy compared to traditional chemically synthesized drugs (Mynarek et al., 2013; Turner and Knechtle, 2013; Pavanello et al., 2017). With patents of innovator biological products expiring, opportunities are opened up for the production of biosimilars that may reach the population at a lower cost and increase patient access to therapy (Kalaivani et al., 2015). In this context, Novex^®^ is the first rituximab biosimilar that has gained marketing authorization for all the approved indications of the innovator product by the Argentinean health authority and other middle-income countries based on analytical quality, nonclinical studies, immunogenicity, and adequate safety in adult patients (Milone et al., 2016; Milone et al., 2017). Emerging evidence suggests that the use of the anti-CD20 antibody rituximab as part of the standard-of-care treatment of hematologic conditions, rheumatic and neurologic diseases, and kidney disorders has shown to be effective and safe in adults (Jung et al., 2014; Chauhan and Mehta, 2019; Abboud et al., 2021; Briani et al., 2021; Narayanaswami et al., 2021). In addition, and due to limited available therapeutic alternatives in a variety of difficult-to-treat conditions in pediatrics, such as immune thrombocytopenia, neuromyelitis optica spectrum disorder, and post-transplant lymphoproliferative disorders, rituximab has been extensively evaluated and proven effective supporting its off-label indications. Nonetheless, pediatric studies on the safety and risk factors related to the development of rituximab-induced adverse drug reactions (ADR) are limited in this age group and mainly derived from studies in adults or small homogeneous pediatric populations (Minard-Colin et al., 2020; McAtee et al., 2021). Similar to other monoclonal antibodies, the main reported rituximab-related ADR are hypersensitivity reactions that emerge due to the direct action of the drug on the immune system or to its intrinsic capacity to enhance an immunological response. The most frequent hypersensitivity reactions to rituximab are infusion-related reactions (IRR), which are defined as temporally associated with the infusion and are generally restricted to the first exposure (Pichler, 2006; Picard and Galvão, 2017; Isabwe et al., 2018; Soyer et al., 2019; Mori et al., 2020). Nonetheless, few reports on the use of rituximab and related ADR are available in pediatrics denoting an urgent need for studies to support the use of biosimilars in children in the context of a pharmacovigilance and risk-management plan to ensure comprehensive care in this age group.

Thus, the aim of this study was to analyze and report evidence of the safety of rituximab (biosimilar Novex^®^ and innovator) used in routine clinical practice and to detect risk factors related to the development of IRR and delayed ADR in a real-life cohort of pediatric patients with complex diseases.

## Materials and Methods

### Study Design and Patient Population

We conducted a prospective study evaluating the active and intensive pharmacovigilance surveillance of rituximab in a single-center cohort at Hospital de Pediatría JP Garrahan (HPG, Buenos Aires, Argentina). The study was approved by the institutional review board (Protocol #1071) and conducted in accordance with the Declaration of Helsinki.

Eligible patients included all children younger than 19 years old treated with rituximab for immunologic (I), hematologic (H), rheumatic (R), neurologic (Neu), or oncologic (O) diseases or transplantation, including solid-organ (SOT) and hematopoietic stem-cell transplantation (HSCT) between March 2019 and February 2020. They were followed-up for 6 months starting at the time of rituximab initiation. Therefore, patients were right censored at 6 months after the first rituximab infusion, last follow-up, death, or introduction of a different chemotherapy regimen in oncology patients, whichever occurred first.

Data were collected from medical records at each visit during rituximab treatment. The frequency of the visits was at the discretion of the treating physician. No additional visits were scheduled as part of the present study. A centralized database with restricted access was generated and the patients included were identified with a unique number. Patients whose medical records were incomplete or not available and patients who were lost to follow-up were excluded from the study.

Patients were not randomly assigned to the innovator or biosimilar rituximab product. The rituximab product (innovator or biosimilar) that patients received at HPG depended on either provision by the health insurance for those who had a health plan or provision by the hospital for those without coverage. In the latter case, the drug product available at HPG depends on a tendering process. Thus, patients were not randomly assigned but received the available drug product.

### Demographic, Clinical, and Biochemical Data

Data retrieved before and after each rituximab infusion included patient demographic and anthropometric information (i.e., age, sex, weight, height), diagnosis, rituximab indication, date, and type of transplant (if applicable), comorbid diseases, and concomitant drug treatments (e.g., steroids or other immunosuppressive drugs). Laboratory data were also recorded and included: white blood cell and absolute lymphocyte counts (ALC), lymphocyte subsets (CD19/CD20 population counts), and biochemical parameters (kidney and liver function tests, electrolytes, total serum gamma globulin levels, and IgA, IgM, and IgG when appropriate).

A detailed list of the different diagnoses of the patients included in the study is provided in Supplementary Table S1. The immune-hemato-rheumatologic conditions (IHR) included pathologies with an immune-mediated mechanism but with no central nervous system involvement (e.g., systemic lupus erythematosus).

### Rituximab Indication and Administration

Rituximab treatment was indicated according to international recommendations, internal consensus of each clinical department, and/or the prescribing information for Argentina (Otte, 2002; Beck et al., 2015), as described in Supplementary Table S2.

The schedule of rituximab administration depended on patient diagnosis and/or indication. A detailed description of the different schedules of drug administration is provided in Supplementary Material (Genberg et al., 2006; Riva et al., 2017; Tenembaum and Yeh, 2020).

During rituximab infusion no other intravenous drugs were administered and vital signs were monitored every 30 min in order to detect the development of IRR to rituximab.

Rituximab infusion-related data included commercial brand (innovator or biosimilar), expiration date and batch number, total dose (mg), final volume of the solution (ml), concentration of the solution (mg/ml), solvent used, and premedication.

### Definition of Adverse Drug Reactions and Severity

An ADR was defined as any harmful event suspected to be caused by rituximab, including hypersensitivity reactions and delayed ADR, detected during this study (Kasi et al., 2012; IBM Micromedex, 2021). ADRs were defined and coded as depicted in Supplementary Table S3 according to the primary System Organ Class (SOC) defined by MedDRA version 17.1, the National Institute of Allergy and Infectious Disease and the Food Allergy and Anaphylaxis Network criteria, the Nathan and Oski’s Hematology and Oncology of Infancy and Childhood, and the Common Terminology Criteria for Adverse Events (CTCAE) v 5.0 (Barten et al., 2006; Manivannan et al., 2009; Orkin et al., 2014; Crépin et al., 2016).

The study of hypersensitivity reactions to rituximab was based on a general classification for monoclonal antibodies consisting of two main types: alpha (including IRR and cytokine release syndrome) and beta (IgE/non-IgE, immune-complex and delayed cell-mediated hypersensitivity reactions) (Mori et al., 2020). Distinguishing alpha from beta hypersensitivity reactions requires specific tests (Isabwe et al., 2018) that were not available in routine clinical practice at our Hospital; therefore, they were not classified. Besides, IRR are the most common hypersensitivity reactions associated with rituximab administration occurring within the first 24 h after infusion (Vogel, 2010; Isabwe et al., 2018). Delayed rituximab-induced ADRs were defined as those elicited between 24 h after the end of infusion and up to 180 days, equivalent to the follow-up period (Vogel, 2010; Kasi et al., 2012; Jung et al., 2014; Lachmann et al., 2017; Legeay et al., 2017; Kamei et al., 2018). In oncology patients, severe hematologic ADR occurring in/outside the cycles of chemotherapy containing rituximab were recorded but not included in the risk analysis due to the impossibility to assess a causality relationship with rituximab due to concomitant chemotherapy.

All suspected ADR were discussed with the treating physician and once confirmed, signs and symptoms, time of onset, infusion rate, and total dose received at the time of the reaction were recorded together with the actions taken by the nurses and physicians (temporary or permanent infusion discontinuation, changes in infusion rate, pharmacological treatment given) and final outcome of the event (resolved, resolved with sequelae, or death).

The causality of the IRR and delayed ADR was assessed by two trained pharmacists and categorized into definite, probable, possible, and unlikely using the Naranjo algorithm (Naranjo et al., 1981).

ADR severity was classified into mild, moderate, severe, or lethal according to WHO classification criteria. In addition, ADR were graded from 1 to 5 based on the v 5.0.

### Statistical Analysis

Continuous variables are reported as median and range and compared using the Student’s t test while categorical variables were compared using the Fisher’s exact test.

The incidence of rituximab-related ADR was calculated as the ratio of the number of patients that developed an event to the total number of patients that received the drug over the study time. Furthermore, the proportion of infusions in which a rituximab-related ADR developed was expressed as the ratio of the number of infusions during which an ADR occurred to the total number of rituximab infusions.

To evaluate risk factors for the development of rituximab-associated IRR and delayed ADR the unadjusted Kaplan-Meier method and the log-rank test were used in univariable analysis with a significance level set at *p*<0.05. For multivariable analysis, a Cox-proportional hazards regression model with stepwise selection was used with a significance level set at *p* < 0.05. Previous to multivariable analysis, we controlled for potential effect modifications and confounders among the variables retained in univariable analysis and interactions between variables in multivariable analyses were tested using the χ2 test.

ROC curves were developed in order to determine the predictive power of the variables that reached significance in the multivariable model. The proportionality criteria of the final models were verified using the Martingale residuals method.

Statistical analysis and graphs were performed using GraphPad Prism v.5., R software and RStudio Version 1.3.959, 2020, Inc (Scalea et al., 2015).

## Results

### Population- and Infusion-Related Characteristics

The study cohort consisted of 77 patients, of whom 57% were female. Median age of the patients was 11.8 years (range, 1.6–18.5) at the start of the study. Rituximab was prescribed for different indications according to each diagnostic group, as shown in Table 1. According to diagnosis, the study population consisted of patients with Neurological (Neu) diseases (*n* = 19), immune-hematologic-rheumatic (IHR) diseases (*n* = 24), solid-organ transplantation (SOT) (*n* = 20), and oncologic (O) diseases-hematopoietic stem-cell transplantation (HSCT) (*n* = 14) (see Supplementary Table S1 for the full list of diagnoses). In IHR patients, rituximab was most frequently prescribed because of refractoriness to first-line therapy (*n* = 18, 75%), while in SOT recipients it was used as induction therapy in sensitized patients (*n* = 9, 42.9%). For patients with neurological diseases, rituximab was mainly indicated as relapse prevention treatment (*n* = 13, 68.4%) and in patients with oncologic diseases, it was mostly used as first-line treatment for high-risk malignancies (*n* = 8, 61.5%). Complete demographic and clinical data for the different diagnostic groups are listed in Table 2. Co-medication information is available in Supplementary Table S4.

**TABLE 1:** Rituximab indications according to disease group.

Disease group	Indication for rituximab	Results (%)
Neurologic diseases (*n* = 19)	Relapse prevention	13 (68.4)
	Refractoriness to first-line treatment	6 (31.6)
Solid-organ Transplantation (*n* = 20)	Immunosuppressive induction	9 (42.9)
	Antibody mediated rejection	7 (33.3)
	Nephrotic syndrome relapse prevention	2 (9.5)
	Cell-mediated rejection	1 (4.8)
	Latent EBV reactivation	1 (4.8)
	PTLD Treatment	1 (4.8)
Immune-hematologic-rheumatic diseases (*n* = 24)	Refractoriness to first-line treatment	18 (72.0)
	Latent EBV reactivation	3 (12.0)
	Autoimmune thrombocytopenia	1 (4.0)
	EBV-positive lymphocytic interstitial pneumonia	1 (4.0)
	First-line treatment	1 (4.0)
Oncologic diseases and HSCT (*n* = 14)	First-line treatment for advanced stage of the disease	8 (61.5)
	Latent EBV reactivation	4 (30.8)
	Pre-transplant conditioning	1 (7.7)

AbbreviationsEBV: Epstein-Barr virus; PTLD: Post-transplant lymphoproliferative disorders; HSCT: hematopoietic stem-cell transplantation.

**TABLE 2:** Demographic and biochemical features of the study population.

Characteristic	Overall	Neu	SOT	IHR	O-HSCT
Number of patients	77	19	20	24	14
Sex (girls/boys)	44/33	11/8	14/6	16/8	3/11
Age(years)	11.8 (1.6–18.5)	13.0 (1.9–17.4)	13.0 (2.7–18.5)	10.2 (1.6–17.5)	9.0 (3.8–16.6)
Weight (kg)	33.2 (7.0–100.0)	43.0 (12.0–100.0)	34.2 (14.3–62.5)	25.6 (7.0–78.0)	32.9 (11.8–65.0)
BSA(m^2^)	1.1 (0.4–2.0)	1.3 (0.5–2.0)	1.2 (0.6–1.7)	1.0 (0.4–1.9)	1.1 (0.6–1.7)
Height (cm)	134 (71–165)	144 (87–160)	142 (91–165)	127 (71–164)	132 (104–165)
Laboratory parameters	SCR creatinine (mg/dl)	0.6 (0.4–0.9)	2.1 (0.3–13.1)	0.6 (0.2–0.8)	0.5 (0.3–1.0)
	Uremia (mg/dl)	24 (13–42)	77 (23–233)	25 (16–72)	29 (11–65)
	Total bilirubin (mg/dl)	0.2 (0.1–1.2)	0.3 (0.3–7.1)	0.3 (0.3–30.6)	0.3 (0.3–0.7)
	AST (U/L)	19 (9–32)	26 (9–315)	26 (14–88)	22 (9–170)
	ALT (U/L)	12 (8–82)	30 (9–650)	27 (8–124)	42 (12–250)

Abbreviations: AST, Aspartate transaminase; ALT, Alanine transaminase; BSA, body surface area; HSCT, Hematopoietic stem-cell transplantation; IHR, Immune-hematologic-rheumatic; Neu, neurologic diseases; O, oncologic diseases; SCR, serum creatinine; SOT, solid-organ transplantation.

Data are shown as median (range).

Almost all patients were followed for 180 days except in nineteen patients due to death because of non-rituximab-related causes (*n* = 4), progression to a different chemotherapy regimen (*n* = 8), or loss to follow-up (*n* = 7).

During the study period we evaluated 187 infusions. The complete data regarding the rituximab infusions are shown in Table 3. Biosimilar rituximab (Novex^®^) was administered in 83% (*n* = 155) of the cycles. One-third of the administrations were first infusions (32.6%, *n* = 61/187). The most commonly used diluent was 5% dextrose (in 58% of all infusions), and the median rituximab dosage was 375 mg/m^2^ (range, 202.4–783.1).

**TABLE 3:** Characteristics of rituximab infusions.

Characteristic	Neurologic diseases (*n* = 19)	Solid-organ transplantation (*n* = 20)	IHR diseases (*n* = 24)	Oncologic diseases and HSCT (*n* = 14)
Total number of infusions	47	37	51	52
Innovator/Biosimilar	7/40	6/31	12/39	7/45
Number of first infusions (%)	11 (23.4)	20 (54)	17 (33.3)	13 (25)
Dose^a^ (mg/m^2^)	384.6 (333.3–750.0)	375 (285.7–416.7)	401.8 (340.0–783.1)	375 (202.4–468.7)
Concentration^a^ (mg/ml)	1.0 (0.6–3.2)	1.0 (0.5–2.0)	1.3 (0.5–2.0)	1.7 (0.7–2.6)
Inpatient/outpatient	18/29	36/1	48/3	34/18
Diluent (5% dextrose/normal saline solution)	14/33	19/18	23/28	52/0
	(30%/70%)	(51%/49%)	(45%/55%)	(100%/0%)
Volume of infusion				
250 ml	14 (30%)	14 (38%)	14 (27%)	51 (98%)
500 ml	33 (70%)	16 (43%)	37 (73%)	-
Other^b^	—	7 (19%)	—	1 (2%)

Abbreviations: IHR, Immune-hematologic-rheumatic; HSCT, Hematopoietic stem-cell transplantation.

a Data are presented as median (range).

b Fractional infusion bag including 100–600 ml.

All patients received premedication therapy 30 min prior to starting rituximab infusion. A triple-drug therapy of diphenhydramine, hydrocortisone, and acetaminophen (1:1:10) was used in 52.9% of the infusions (*n* = 99), diphenhydramine and hydrocortisone (1:1) in 26.2% (*n* = 46), and diphenhydramine and acetaminophen (1:10) in 18.2% (*n* = 34).

Interestingly, different absolute lymphocyte counts (ALC) were observed in the diagnostic groups before starting rituximab, with higher ALC counts in children with Neu-IHR diseases compared to the O-HSCT-SOT groups (2.180 x 10^3^/mm^3^ *vs.* 0.980 x 10^3^/mm^3^, respectively, *p* < 0.05). Based on these findings we decided to generate the dichotomic variable “type of diagnosis” assigning a value of 0 to O-HSCT-SOT patients, and 1 to patients with Neu-IHR diseases.

### Rituximab Adverse Drug Reactions

**FIGURE 1:**
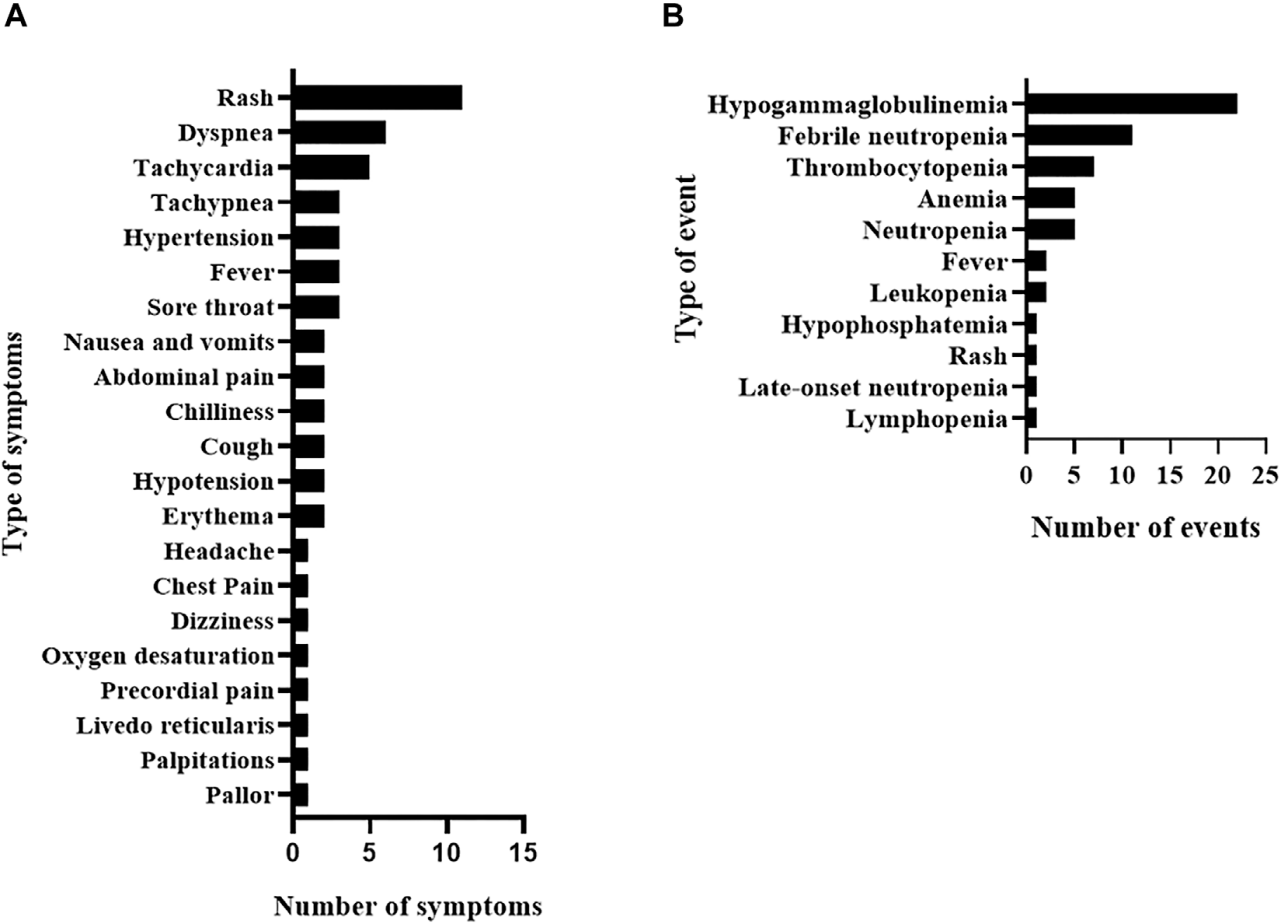
Rituximab adverse drug reactions in the study population including infusion-related reactions and their associated symptoms **(A)** and delayed adverse drug reactions **(B)**.

Overall, 87 ADR, consisting of 29 IRR and 58 delayed ADR, affected 48 of the total 77 studied patients (62.3%). In each IRR, one or more associated symptoms were observed (Figure 1A); the most frequent delayed ADR was hypogammaglobulinemia as shown in Figure 1B.

The proportion of infusions in which rituximab innovator and biosimilar-related ADR developed was 25.0 and 38.7%, respectively. No difference was observed in the proportion of infusions with IRR, severe IRR, delayed ADR, and severe delayed ADR when comparing innovator and biosimilar rituximab (Fisher’s exact test, *p* > 0.05; Table 4). However, this observation may be due to the low sample size of the innovator rituximab group.

**TABLE 4:** Number of infusions with ADR in the study population.

	Biosimilar	Innovator	*p* value
Number of infusions	155	32	
Number of patients	69	25	
Age (years)	11.2 (1.6–18.5)	11.0 (1.6–17.4)	>0.05
Total number of ADRs	60	8	>0.05
IRRs	26	3	>0.05
Severe IRRs	3	0	>0.05
Delayed ADR	34	5	>0.05
Severe delayed ADR	10	1	>0.05

AbbreviationsADR: adverse drug reactions; IRR: infusion related reactions.

### Infusion Related Reactions

**FIGURE 2:**
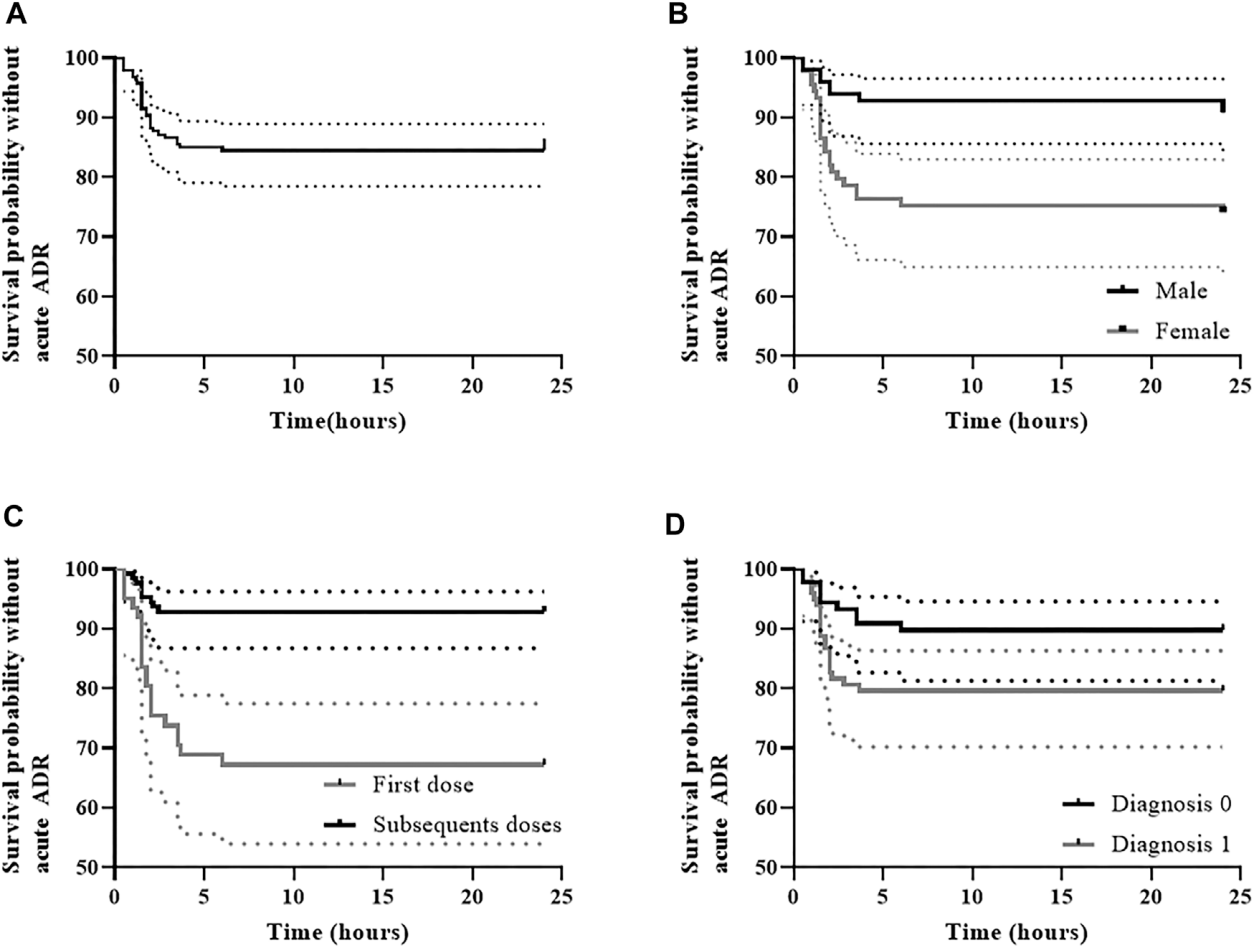
Rituximab infusion-related reaction-free survival **(A)** and IRR-free survival according to the variables retained in multivariable analysis, including **(B)** sex, **(C)** first dose versus subsequent doses, and **(D)** type of diagnosis. **(A)** IRR-free survival was 84.5% (95% CI, 79.5–89.8) at 6 h post-initiation of rituximab therapy; (**B)** In males IRR-free survival was 92.9% (95% CI, 87.9–98.1) at 6 h post-initiation of rituximab therapy, whereas in females it was 75.3% (95%CI, 66.8–84.8); **(C)** IRR-free survival was 67.2% (95% CI, 56.4–80.1) during the first dose at 6 h post-initiation of rituximab therapy, whereas in subsequent doses survival was 92.9% (95% CI, 88.5–97.5); (**D)** In patients with diagnosis 0 (O-HSCT-SOT), IRR-free survival was 89.9% (95% CI, 83.8–96.4) at 6 h post-initiation of rituximab therapy, whereas it was 79.6% (95% CI, 72.0–88.0) in patients with diagnosis 1 (N-IHR).

Rituximab IRR occurred in 15.5% of the infusions (29/187) corresponding to a probability of IRR-free survival of 84% (IC95% 79–89) at 6 h post-initiation of rituximab therapy as shown in Figure 2A. On the other hand, IRR occurred in 35.1% of the patients (27/77), of whom 92.6% (25/27) experienced only one IRR. Most IRR (*n* = 22, 76%) occurred within the first 2 h after rituximab administration with a median time of onset of 1.5 h (range, 0.5–6.0). Interestingly, 69% of the IRR developed during the first rituximab infusion (20/29) compared to only 7% in subsequent infusions. Nonetheless, in only one-third of the total first infusions (20/61) an IRR to rituximab occurred.

Regarding severity, 79.3% of the IRR were classified as moderate (23/29), 10.3% as mild (3/29), and 10.3% as severe (3/29) according to WHO criteria. In addition, IRR were grades 1/2 and 3/4 of the CTCAE in 72.4% (21/29) and 27.6% (8/29), respectively. No sequelae or death related toxicity were reported for any of the IRR after rituximab discontinuation.

In causality assessment, 89.7% of the IRRs were probably (26/29), 6.9% were possibly (2/29), and 3.4% were definitely related to rituximab (1/29).

When evaluating diagnosis, 37.9% of the IRR occurred in IHR patients (11/29), 31.0% in Neu patients (*n* = 9/29), 20.7% in SOT recipients (6/29), and 10.3% in O-HSCT patients (*n* = 3/29).

Overall, 54 different signs and symptoms were observed in all 29 IRR. Respiratory symptoms, such as dyspnea, tachypnea, and sore throat, were the most commonly observed (*n* = 16, 29.6%; Table 5) developing in 9 IRR (one IRR may account for 4 different respiratory symptoms as depicted in Table 5 and Supplementary Figure S1) followed by skin and subcutaneous symptoms (*n* = 15, 27.8%), including rash and erythema (full list is shown in Table 5). Most IRR were associated with only one symptom but in eight events, more than one organ system was affected (Supplementary Figure S1).

**TABLE 5:** Infusion-related reactions to rituximab: signs and symptoms by affected organ system.

Signs and symptoms	Number (%)	Biosimilar (%)	Innovator (%)
**Skin and subcutaneous tissue symptoms**	**15 (27.8)**	**13 (25.5)**	**2 (66.7)**
Rash	11 (20.4)	9 (17.6)	2 (66.7)
Erythema	2 (3.7)	2 (3.9)	—
Pallor	1 (1.9)	1 (2.0)	—
Livedo reticularis	1 (1.9)	1 (2.0)	—
**Cardiovascular symptoms**	**12 (22.2)**	**12 (23.5)**	**0**
Tachycardia	5 (9.3)	5 (9.8)	—
Hypertension	3 (5.6)	3 (5.9)	—
Hypotension	2 (3.7)	2 (3.9)	—
Precordial pain	1 (1.9)	1 (2.0)	—
Palpitations	1 (1.9)	1 (2.0)	—
**Respiratory, chest symptoms**	**16 (29.6)**	**16 (31.4)**	**0**
Dyspnea/ Breathing difficulties	6 (11.1)	6 (11.8)	—
Sore throat	3 (5.6)	3 (5.9)	—
Tachypnea	3 (5.6)	3 (5.9)	—
Coughing and associated symptoms	2 (3.7)	2 (3.9)	—
Chest pain	1 (1.9)	1 (2.0)	—
Oxygen desaturation	1 (1.9)	1 (2.0)	—
**Nervous system symptoms**	**4 (7.4)**	**3 (5.9)**	**1 (33.3)**
Chilliness	2 (3.7)	2 (3.9)	-
Headache	1 (1.9)	-	1 (33.3)
Dizziness	1 (1.9)	1 (2.0)	—
**Gastrointestinal symptoms**	**4 (7.4)**	**4 (7.8)**	**0**
Abdominal pain	2 (3.7)	2 (3.9)	—
Nausea and vomiting	2 (3.7)	2 (3.9)	—
**General symptoms**	**3 (5.6)**	**3 (5.9)**	**0**
Fever	3 (5.6)	3 (5.9)	—
**Total symptoms**	**54 (100)**	**51 (100)**	**3 (100)**

In all cases, actions taken after the development of IRR included temporary interruption of the infusion, administration of steroids and/or diphenhydramine, and/or reduction of infusion rate. In all but two patients, IRR completely resolved after the rituximab infusion was interrupted and restarted at a slower rate. In these two patients, treatment was permanently discontinued due to a severe IRR, consisting of pruritic morbilliform rash in one and anaphylactic shock in the other. Actions taken after development of IRR are described in Supplementary Table S5.

First *versus* subsequent infusions (HR 5.4, IC 95% 2.4–12.1, *p*˂0.05) and diagnosis type 1 *versus* 0 (Neu-IHR diseases *vs.* O-HSCT-SOT, HR 2.3, IC 95% 1.02–5.4, *p* < 0.05) were associated with an increased risk of IRR in the final Cox multivariable model (Table 6; Figures 2C,D). On the other hand, male patients were found to be at a lower risk of developing IRR (HR 0.3, IC 95% 0.1–0.8, *p*<0.05) (Figure 2B).

**TABLE 6:** Univariable and multivariable analysis for the development of IRR and delayed ADR to rituximab in the study population.

Infusion-related reactions	Univariable analysis	*p-value*	Multivariable analysis	*p-value*
Factor	Hazard ratio (95% CI)		Hazard ratio (95% CI)	
First *vs.* subsequent doses	5.226 (2.487–10.980)	<0.001	5.423 (2.431–12.097)	<0.001
Sex 1 *vs.* 0 (1: male; 0: female)	0.264 (0.119–0.582)	<0.001	0.341 (0.146–0.796)	<0.05
Dose (mg)	1.002 (1.000–1.003)	<0.05	-	NS
Accumulated BSA-normalized dosage (mg/m^2^)	1.002 (0.999–1.004)	0.124	-	NS
Body weight (kg)	1.021 (1.002–1.042)	<0.05	-	NS
Age (years)	1.066 (0.968–1.175)	0.195	-	NS
Diagnosis 1 *vs.* 0	2.158 (0.979–4.760)	0.057	2.333 (1.016–5.359)	<0.05
Delayed ADR	Univariable analysis		Multivariable analysis	
Factor	Hazard Ratio (95% CI)	*p-value*	Hazard Ratio (95% IC)	*p-[value*
Accumulated BSA-normalized dosage (mg/m^2^)	1.0002 (1.0001–1.0007)	<0.05	1.0003 (1.0001–1.0006)	<0.05
First dose *vs.* subsequent doses	0.357 (0.128–0.994)	<0.05	-	NS
Diagnosis 1 *vs.* 0	0.445 (0.214–0.927)	0.030	0.401 (0.183–0.883)	<0.05
BSA (m^2^)	1.72 (0.820–3.625)	0.151	-	NS

Abbreviations: BSA, body surface area; CI, confidence interval; Diagnosis 0, solid-organ transplantation and oncological diseases and hematopoietic stem-cell transplantation; Diagnosis 1, neurologic diseases and immune-hematologic-rheumatic disease; NS: not significant

### Delayed Adverse Drug Reactions

**FIGURE 3:**
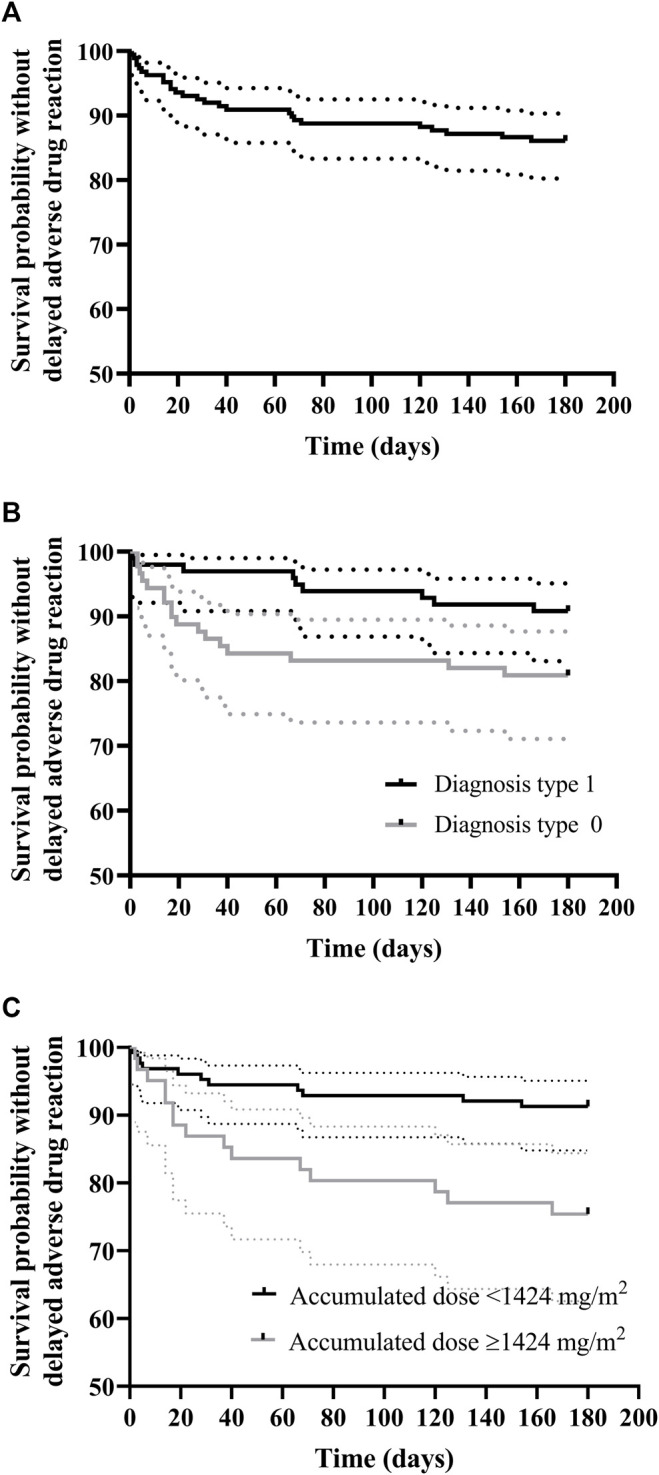
Kaplan–Meier curve for rituximab delayed adverse drug reaction-free survival **(A)** and survival according to **(B)** diagnosis and **(C)** cumulative dose normalized by body-surface area (mg/m^2^). **(A)** Delayed ADR-free survival was 86.1% (95% CI, 81.3–91.2) at 166 days post initiation of rituximab therapy; **(B)** Delayed ADR-free survival for diagnosis 0 (O-HSCT-SOT) was 80.9% (95% CI, 73.1–89.5) at 154 days post-initiation of rituximab therapy, whereas for diagnosis 1 (NIHR) it was 90.8% (95%CI, 85.3–96.7) at 166 days; **(C)** Delayed ADR-free survival for patients with cumulative dose normalized by body surface area (mg/m^2^) ≥ 1,424 mg/m^2^ was 81.1% (95% CI, 74.5–88.4) at 166 days post initiation of rituximab therapy, whereas for patients with cumulative dose normalized by body-surface area (mg/m^2^) < 1,424 mg/m^2^ it was 95.4% (95% CI, 90.4–1.00) in the study period.

Rituximab-related delayed ADR (*n* = 58) developed after 23.0% of the infusions (43/187) and in 37.7% of the patients (29/77) at a median time of 7 days (range, 1–166) after rituximab administration. The probability of delayed ADR-free survival was 86.1% (CI95% 81.3–91.2) at 166 days after initiation of rituximab therapy (Figure 3A). Patients with cancer were most commonly affected (63.8%) by these adverse events, followed by both neurological and SOT patients (13.8%), and IHR (8.6%).

As shown in Table 7 the most frequently observed ADR was hypogammaglobulinemia accounting for 37.9% of all delayed ADR, with an incidence of 28.6% (in 22/77 patients). Hypogammaglobulinemia manifested as a median decrease in blood gammaglobulin of 46.3% (range, 5.1–90.6%). Particularly for the immunoglobulins (Ig), the median decrease from the lower normal value for IgG was of 39.3% (range, 5.1–63.9%), for IgA was 44.6% (range, 11.4–90.6%), and for IgM was 50.0% (range, 15.4–80.0%). In 15/22 events of hypogammaglobulinemia a decrease in IgG (68.2%), in thirteen events a decrease in IgA (59.1%), in eight events a decrease in IgM (40%), and in fifteen events a decrease in all three immunoglobulins (68.2%) was observed. Median time from rituximab initiation to onset of hypogammaglobulinemia was 33.0 days (range, 3.0–166.0) and intravenous immunoglobulin (IVIG) was given after ten events. One patient with juvenile dermatomyositis had persistently low gammaglobulin levels for 12 months requiring 12 monthly infusions of IVIG.

**TABLE 7:** Delayed adverse reactions to rituximab according to the affected system (n = 58).

Adverse events classified by organ system (n = 31)	Number of events (%)
**Skin and subcutaneous tissue**	**1 (1.7)**
Rash	1 (1.7)
**Immune system**	**22 (37.9)**
Hypogammaglobulinemia	22 (37.9)
**Blood and lymphatic system**	**5 (8.6)**
Lymphopenia	1 (1.7)
Neutropenia^a^	1 (1.7)
Late-onset neutropenia	1 (1.7)
Leukopenia	2 (3.4)
**General**	**2 (3.4)**
Fever	2 (3.4)
**Metabolism and nutrition**	**1 (1.7)**
Hypophosphatemia	1 (1.7)
**Severe blood and lymphatic system disorders with shared causality in immunosuppressed patients with oncological diseases, HSCT, and solid-organ transplantation (n = 27)**	
Neutropenia	4 (6.9)
Thrombocytopenia	7 (11.3)
Anemia	5 (8.6)
Febrile neutropenia	11 (21.2)
**Total events**	**58 (100)**

Only severe (grade 3/4 blood and lymphatic adverse reactions according to the CTCAE) are shown.

a Corresponds to a grade 3 CTCAE, severity grading scale in a neurologic patient with NMOSD MOG+.

Abbreviations: HSCT, Hematopoietic stem-cell transplantation.

According to the WHO severity classification, 36.2% (21/58) of the delayed ADR were mild, 34.5% (20/58) were moderate, and 29.3% (17/58) were severe. Overall, 35 delayed ADR were evaluable using the CTCAE severity grading scale, excluding hypogammaglobulinemia since it is not specified in this database. Of this subset of events, most were grade 3/4 (28/35, 80.0%) corresponding to febrile neutropenia and thrombocytopenia requiring blood transfusions. Twenty-seven of these delayed ADR may also have been due to other immunosuppressive or antineoplastic drugs that increase the risk of severe delayed ADR, such as myelosuppression (e.g., etoposide, vincristine, methotrexate, sirolimus, tacrolimus, and mycophenolate) (Table 7).

Excluding the 27 events of delayed ADR that developed in oncology, HSCT, and solid-organ transplant patients (severe hematologic ADR occurring in/outside the cycles of chemotherapy containing rituximab), 31 events were recorded and 26 of them were the first event in each patient. Thus, 26 delayed ADRs, of which 20 consisted of hypogammaglobulinemia (77%), were analyzed in the risk assessment.

The unadjusted Kaplan–Meier curves for delayed ADR-free survival according to significant risk factors are shown in Figures 3B,C. Briefly, and contrary to IRR risk factors, patients with a type 1 diagnosis (Neu-IHR diseases) were at a 60% lower risk of delayed rituximab-related ADR than those with a type 0 diagnosis (O-HSCT-SOT; HR 0.4, IC 95% 0.18–0.88, *p* = 0.023) as shown in Table 6. In addition, there was a 3% increased risk of a delayed ADR with every 100 mg/m^2^ of the cumulative body surface area (BSA)-normalized dosage (HR 1.0003, 95% CI, 1.0001–1.0006, *p* = 0.041). In this sense, the ROC curve yielded an area under the curve of 0.65 (95% CI, 0.54–0.76) for the cumulative BSA-normalized dosage. The Youden index demonstrated that a cumulative BSA-normalized dosage >1,424 mg/m^2^ was the optimal cut-off for the prediction of a delayed ADR (specificity 0.714 and sensitivity 0.577).

## Discussion

In the present study, we developed and implemented an intensive pharmacovigilance program in order to evaluate the safety profile of the use of rituximab (biosimilar Novex^®^ and innovator) in the real-life follow-up of a large pediatric population diagnosed with rare and complex diseases. The incidence of rituximab-related ADRs was as expected based on previous studies evaluating the use of innovator rituximab in children and adults (Kasi et al., 2012; Jung et al., 2014; Milone et al., 2016; Legeay et al., 2017; Milone et al., 2017; Kamei et al., 2018; Minard-Colin et al., 2020; McAtee et al., 2021). Most IRR (70%) occurred during the first infusion. Nonetheless, in line with previous reports, IRR occurred only in one-third of the infusions (Jung et al., 2014; Legeay et al., 2017; Levin et al., 2017). Peri-treatment factors associated with an increased risk of developing rituximab-related IRR were first infusion, being female, and Neu-IHR diagnosis, whereas those associated with an increased risk of delayed ADRs were O-HSCT-SOT diagnosis and cumulative BSA-normalized dosage.

Of note, almost all but one patient of our study population received rituximab as an off-label prescription for more than 20 different indications. This shows the widespread use of rituximab in children with rare diseases and highlights the key role of active pharmacovigilance in special populations with off-label prescriptions of rituximab and limited reports.

The pattern of adverse events found in our study is consistent with previous studies in children and adults. In our study, the incidence of rituximab-related IRR was 15%, similar to a recent publication in a heterogeneous pediatric population and adults with B-cell cancers receiving the innovator drug (Jung et al., 2014; Legeay et al., 2017). Nevertheless, our results differ from those of previous studies in children with cancer and nephrotic syndrome showing an incidence of rituximab-related IRR ranging from 53 to 80% (Maloney et al., 1997; McLaughlin et al., 1998; Piro et al., 1999; Davis et al., 2000; Lenz, 2007; Kamei et al., 2018). This difference may be explained by the inclusion criteria used in those study populations (complicated nephrotic syndrome) and the exclusion of rituximab infusions during B-cell depletion (i.e., subsequent cycles after the first rituximab infusion), as the incidence of IRR is much lower during B-cell depletion decreasing the incidence of IRR in our study. In addition, similarly to a retrospective study evaluating a heterogeneous population of children and young adults in whom 72% of IRR occurred during the first dose. (McAtee et al., 2021), we found that 69% of IRR developed during the first infusion. This result emphasizes the need for rigorous surveillance of monoclonal antibodies in children, especially at the first administration.

When evaluating the organ systems affected by rituximab-induced IRR (Table 5 depicts the signs and symptoms of the registered IRR, *n* = 54, by affected organ system and Supplementary Figure S1 depicts the organ systems affected in each infusion, *n* = 29, that an IRR occurred), our results partially correlate with previous findings (Legeay et al., 2017). As expected, the skin was the main organ system affected by IRR during rituximab administration in our cohort (Supplementary Figure S1). Those IRR may include many cutaneous symptoms as depicted in Table 5. Nevertheless, severe ADR, such as Stevens-Johnson syndrome or toxic epidermal necrolysis were not detected in our series. In our study, cardiovascular symptoms, including tachycardia, hypotension, and hypertension were the second most common IRR and in line with reports in adults (Brennan et al., 2009; Kamei et al., 2018). The third most common IRR were respiratory tract symptoms in agreement with international databases, the package insert of innovator rituximab, and previous studies in children and adults (32–80%) (Otte, 2002; Brennan et al., 2009; Legeay et al., 2017). Also, gastrointestinal symptoms including vomiting, nausea, and abdominal pain were encountered at incidences similar to data in literature (Jung et al., 2014; Legeay et al., 2017). Finally, in our study the incidence of general symptoms, was lower than prior studies in which other symptoms were reported including peripheral edema, asthenia, and physical deterioration (Jung et al., 2014). The lower incidence of this effect may be a result of the use of premedication including antipyretics. Finally, CNS IRR occurred in a low proportion of patients (Supplementary Figure S1).

As expected, we observed that almost 80% of IRR were mild or moderate according to both the WHO criteria and the CTCAE. Nonetheless, we also recorded three severe reactions consisting of hypersensitivity, pruritic morbilliform rash, and anaphylactic shock that developed in patients with juvenile dermatomyositis, juvenile idiopathic arthritis, and systemic lupus erythematosus, respectively. Therefore, we registered an incidence of anaphylaxis of 1.3%, which is lower than that found in a previous report (McAtee et al., 2021). Interestingly, IHR patients had IRR with more diverse symptoms (two or more) affecting different organs compared to the other diagnostic groups (Supplementary Figure S1). Furthermore, in IHR patients a higher proportion of grade 3/4 IRR (55%) was seen, compared to patients with other diagnoses (Neu, 25%; SOT, 16.7%; O-HSCT, 0%). A possible explanation is based on a higher ALC value of IHR patients and this elevated ALC values have been identified as a risk factor for developing IRR in other populations (Lang et al., 2013). In addition, a high ALC due to the immune response associated with the underlying disease may also play a role in the severity of the IRR-associated symptoms observed in this group of patients (Winkler et al., 1999; Lang et al., 2013).

### Risk Factors for IRR

The identification of risk factors for IRR is important to optimize rituximab treatment and minimize the occurrence of ADR. In our study, the first rituximab infusion and type 1 diagnosis (Neu-IHR) were positively correlated with the development of IRR. Similar to other studies (Kamei et al., 2018; Soyer et al., 2019), our patients were found to have a 5-fold higher risk of developing IRR during the first infusion of rituximab than in subsequent cycles. In this sense, Legeay et al. observed a 39% higher risk of IRR during the first exposure to the monoclonal antibody and McAtee et al. reported that the odds of IRR decreased with successive doses (Kamei et al., 2018; McAtee et al., 2021). Monoclonal antibodies release proinflammatory cytokines, such as IL-6 and TNF-alpha, from target cells during the first 10 min to 24 h after starting the infusion and usually during the first administration (Winkler et al., 1999; Calogiuri et al., 2008; D’Arena et al., 2017). The exact mechanism for this is unknown, although cytokine release might play a significant role in IRR as these reactions are related to an increase in the serum concentration of pro-inflammatory cytokines (e.g., IL-6 and TNF-α) mediated by both target (B cells) and effector (NK cells and macrophages) cells (Winkler et al., 1999; Wing, 2008; Jones et al., 2014; Puxeddu et al., 2016). This hypothesis is also sustained by the findings of higher IL-6 levels in patients who received rituximab and developed a hypersensitivity reaction (Winkler et al., 1999; Isabwe et al., 2018). In addition, to further evaluate the association between rituximab and first-infusion IRR, we analyzed a subcohort of 61 first infusions. In this sub-analysis, patients with lymphopenia, were at a lower risk of IRR than those with ALC within the reference range (HR 1.2, 95% CI, 1.02–1.5, *p* value = 0.034). The corresponding receiver-operating characteristics (ROC) curve (AUC 0.593, CI95% 0.442–0.743) yielded a Youden index of 680/mm^3^ as the cut-off value for ALC that best discriminated the development of a first-infusion IRR.

In our study, type of diagnosis was also a risk factor associated with rituximab IRR, as patients with Neu-IHR diseases had a 133% higher risk of developing IRR than patients with O-HSCT-SOT conditions. This finding may be explained by differences in rituximab doses and ALC between groups. Patients with Neu-IHR diseases receive higher doses of rituximab than those with O-HSCT-SOT diseases (500–750 mg/m^2^ *vs.* 375 mg/m^2^), although dosage was not significantly associated with IRR probably due to the low sample size. In addition, Neu-IHR diseases have an immunoreactive component that could be partially explained by the higher ALC as a surrogate for a larger target cell population resulting in an increased release of pro-inflammatory cytokines.

The third variable associated with IRR in the risk analysis was sex, as boys were found to be at a 66% lower risk of developing IRR than girls. In an adult population, Jung et al. described that the subpopulation that suffered at least one adverse reaction consisted of a smaller proportion of men (Jung et al., 2014). In addition, females may be at a higher risk of developing IRR as a result of higher rituximab systemic exposure due to slower clearance compared to males (Riedl and Casillas, 2003; Müller et al., 2012).

### Delayed Adverse Drug Reaction

Overall, 58 delayed ADR were observed after the administration of 43 infusions (23.0%, 43/187). A frequent immune disorder in our cohort of patients was hypogammaglobulinemia probably related to rituximab-induced depletion of the pre-plasma B-cell population (Wunderlich et al., 2017), with an incidence of 28.6%, similar to previous reports in children (McAtee et al., 2021). In a large study in adult patients with rheumatoid arthritis, van Vollenhoven et al. reported an incidence of hypogammaglobulinemia of 24% with IgM and IgG below the normal values for at least 4 months after the last cycle of rituximab (van Vollenhoven et al., 2015). Nonetheless, our results are lower than the 56% reported for an oncologic pediatric population, probably reflecting the importance of the role of the condition at baseline in the development of ADR.

According to VigiLyze global database of adverse events (data provided upon request), blood and lymphatic system disorders, including neutropenia, febrile neutropenia, anemia, and thrombocytopenia, occurred in 18.3% of the patients, in line with the incidence found in our study (18.2%, 14/77). Moreover, in our study grade 3/4 febrile neutropenia was observed in 7.8% in agreement with other reports in pediatric oncology (11.7%) (Minard-Colin et al., 2020). Nonetheless, these results should be interpreted in the context of simultaneous multiple chemotherapy treatments that may synergize the hematological toxicity potentially related to rituximab.

### Risk Factors for Delayed ADR

In our patients with N-IHR diseases the risk of developing a delayed ADR was 60% lower than in patients with O-HSCT-SOT conditions. Similarly, McAtee et al. found that the risk of hypogammaglobulinemia was 2-fold higher and the risk of neutropenia 6-fold higher in patients with cancer than in those with other conditions at baseline (McAtee et al., 2021).

In our study, the reason for the difference between the two diagnostic groups may be associated with the standard concomitant medication received by each group. Prolonged peripheral B-cell depletion induced by immunosuppressive drugs and/or chemotherapy used concomitantly with rituximab may contribute to hypogammaglobulinemia and the suppression of protective antibodies (van Vollenhoven et al., 2015; Cortazar et al., 2017). However, data on the effects of frequently used immunosuppressive drugs on serum immunoglobulins in different conditions could not be elucidated in our study due to the small sample size.

A statistically significant association was observed between the cumulative BSA-normalized dosage and the development of delayed ADR. We found that a cut-off value of 1,424 mg/m^2^ best predicted the cumulative dosage leading to hypogammaglobulinemia. This finding is consistent with that of others who identified the association between the increasing number of rituximab doses and the development of cytopenia and hypogammaglobulinemia (Cattaneo et al., 2006; Boleto et al., 2018).

In order to avoid hypogammaglobulinemia or reduce prolonged deficiency, regulated administration of IVIG after the second dose may be recommended. This is especially important considering the long half-life of IVIG (30 days) and the long courses required to lead to a benefit (Ochs et al., 2018). Other recommendations include antibiotic prophylaxis in patients with pre-existing hypogammaglobulinemia or respiratory diseases. Nevertheless, data are limited and strategies to reduce infections following rituximab administration should be studied prospectively, particularly regarding the combined use of IVIG (McAtee et al., 2021).

Our study has the advantage of reflecting real-world clinical practice at a pediatric tertiary referral hospital and the results are supportive of the use of Novex^®^ in children. Nevertheless, some limitations should be acknowledged. First, although we reliably recorded the rituximab brand our patients received, infusions with innovator rituximab are underrepresented hampering comparison of adverse event rates between biosimilar and innovator rituximab. Second, due to the unavailability of tests we were unable to distinguish the intrinsic mechanism causing the hypersensitivity reactions. In addition, infectious episodes were not recorded, although the clinical impact of this delayed ADR could be determined based on IVIG requirements. Finally, the overall lymphocyte population was measured without distinguishing CD20-expressing B cells susceptible to the action of rituximab because of unavailable consistent routine laboratory tests. Nonetheless, we are confident of the quality of the data based on the prospective and intensive nature of the collection process.

Altogether, to our knowledge this is the first prospective study assessing the incidence of rituximab IRR and delayed ADR as well as associated risk factors in a large heterogeneous pediatric population treated with biosimilar and innovator rituximab. Our study is the first description of the conditions for which rituximab is currently used in the off-label treatment of pediatric patients with challenging diseases in Latin America and may be a major step toward improving access to biologics in the region. In addition, the results of this study support the findings of an earlier analysis in adult patients treated with Novex^®^ and provide evidence that biosimilar rituximab is safe in children with a range of complex diseases (Milone et al., 2016; Milone et al., 2017). Nonetheless, further studies are necessary to detect new safety signals or uncommon severe adverse events with a strong role for active pharmacovigilance in children treated with these off-label biological products.

## Data Availability

The original contributions presented in the study are included in the article/Supplementary Material, further inquiries can be directed to the corresponding authors.

## Abbreviations

ADEM, acute disseminated encephalomyelitis; ADR, adverse drug reactions; ALC, absolute lymphocyte count; BSA, body surface area; CI, confidence interval; CMV, cytomegalovirus; EBV, epstein-barr virus; GPA, granulomatosis with polyangiitis; H, hematological disease; HPG, hospital de pediatrìa garrahan; HR:, hazard ratio; HSCT, hematopoietic stem-cell transplantation; I, immunologic disease; Ig, immunoglobulin; IHR, immune-hematologic-rheumatic diseases; IRR, infusion related reactions; MPA, microscopic polyangiitis; Neu, neurologic disease; NMOSD, neuromyelitis optica sprectrum disorder; O, oncologic disease; R, rheumatic disease; ROC, receiver-operating characteristics; SOT, solid-organ transplantation.
