# Supplementary material for: Intensive Safety Monitoring of Rituximab (Biosimilar Novex® and the Innovator) in Pediatric Patients With Complex Diseases
Source: Front Pharmacol. 2022 Jan 26;12:785770. doi: 10.3389/fphar.2021.785770 (PMC8827405; doi:10.3389/fphar.2021.785770)
Supplement: Supplementary file 1 [file DataSheet1.doc]

**Supplementary materials: Intensive Safety Monitoring of Rituximab (Biosimilar Novex® and the Innovator) in Pediatric Patients with Complex Diseases**

Natalia Riva^1, 2*†^, Manuel Molina^1*†^, Berta L. Cornaló^1*^, María V. Salvador^3^, Andrea Savransky^4^, Silvia Tenembaum^4^, María M. Katsicas^5^, Marta Monteverde^6^, Paulo Caceres Guido^3,7^, Marcela Rousseau^8^, Raquel Staciuk^9^, Agustín González Correas^9^, Pedro Zubizarreta^10^, Oscar Imventarza^11^, Eduardo Lagomarsino^3^, Eduardo Spitzer^12^, Marcelo Tinelli^12^, Paula Schaiquevich^1,2^.

**Supplementary materials index**

1. **Supplementary methods**

**Rituximab administration (page 2)**

1. **Supplementary Figures**

Supplementary Figure 1. Organ systems affected by each of the detected IRR (page 3)

1. **Supplementary Tables**

Supplementary Table 1. Diagnoses of the study population (n = 77). (page 4)

Supplementary Table 2. Rituximab infusion regimen based on patient diagnosis and/or indication. (page 5, 6)

Supplementary Table 3. Operational definition of adverse drug reactions induced by rituximab. (page 7, 8)

Supplementary Table 4. Concomitant medication received by the study population. (page 9)

Supplementary Table 5. Actions taken after the development of IRRs according to symptomatology. (page 10)

**Supplementary methods. Rituximab administration**

The initial infusion rate of rituximab was 50 mg/h and, in the absence of toxicity, infusion rate was increased to 100 mg/h. Thereafter, the rate was increased by 50 mg/h increments every 30 minutes to a maximum of 400 mg/h if tolerated. The infusion regimen for non-Hodgkin lymphoma and leukemia patients was based on the dosing regimen recommended by the Berlin-Frankfurt-Münster (BFM) 95-based protocol combined with rituximab (R+BFM95). Patients with neurologic diseases received rituximab according to internal consensus, (Arroyo, 2019; Tenembaum and Yeh, 2020) those with rheumatic diseases based on international guidelines, (Riva et al., 2017) and kidney and liver transplant recipients based on previous reports. (Genberg et al., 2006) In all cases, patients received premedication consisting of a combination of diphenhydramine, acetaminophen, and/or hydrocortisone.

Supplementary Figure 1. Organ systems affected in each rituximab infusion that an IRR developed in the study cohort

| **# Infusion-Related**  **Reaction *** | | **1** | **2** | **3** | **4** | **5** | **6** | **7** | **8** | **9** | **10** | **11** | **12** | **13** | **14** | **15** | **16** | **17** | **18** | **19** | **20** | **21** | **22** | **23** | **24** | **25** | **26** | **27** | **28** | **29** |
| --- | --- | --- | --- | --- | --- | --- | --- | --- | --- | --- | --- | --- | --- | --- | --- | --- | --- | --- | --- | --- | --- | --- | --- | --- | --- | --- | --- | --- | --- | --- |
| **Condition at baseline** | |  |  |  |  |  |  |  |  |  |  |  |  |  |  |  |  |  |  |  |  |  |  |  |  |  |  |  |  |  |
| Organ system affected | Cutaneous |  |  |  |  |  |  |  |  |  |  |  |  |  |  |  |  |  |  |  |  |  |  |  |  |  |  |  |  |  |
|  | Respiratory |  |  |  |  |  |  |  |  |  |  |  |  |  |  |  |  |  |  |  |  |  |  |  |  |  |  |  |  |  |
|  | Cardiovascular |  |  |  |  |  |  |  |  |  |  |  |  |  |  |  |  |  |  |  |  |  |  |  |  |  |  |  |  |  |
|  | Neurologic |  |  |  |  |  |  |  |  |  |  |  |  |  |  |  |  |  |  |  |  |  |  |  |  |  |  |  |  |  |
|  | Gastrointestinal |  |  |  |  |  |  |  |  |  |  |  |  |  |  |  |  |  |  |  |  |  |  |  |  |  |  |  |  |  |
|  | General |  |  |  |  |  |  |  |  |  |  |  |  |  |  |  |  |  |  |  |  |  |  |  |  |  |  |  |  |  |

*Overall, 29 IRR developed in 27 patients.

Cutaneous symptoms affected 18% of the population treated with rituximab (14/77). Rash was the most frequent symptom (73.3%, 11/15).

Cardiovascular symptoms occurred in 13% of the patients (10/77) and were observed in 34.5% (10/29) of the IRR.

Respiratory symptoms occurred in 11.7% of patients (9/77). These symptoms were observed in 31% (9/29) of IRR.

Gastrointestinal symptoms occurred in 5.2% (4/77) of the patients and accounted for 13.8% (4/29) of the IRR.

General symptoms (fever) developed in 3.9% (3/77) of patients and 10.3% of IRR (3/29).

Neurologic symptoms occurred in 5.2% (4/77) of the patients and 13.8% of the IRR (4/29).

Colors: Yellow: Neurologic diseases; Violet: Solid-organ transplantation; Light blue: Immuno-hematologic-rheumatic diseases; Green: Oncologic diseases.

Supplementary Table 1. Diagnoses of the study population (n=77)

| **Neurologic diseases** | **n=19 (25%)** |
| --- | --- |
| Neuromyelitis optica spectrum disorder | 7 |
| Acute disseminated encephalomyelitis | 3 |
| Autoimmune encephalitis | 2 |
| Opsoclonus myoclonus syndrome | 2 |
| Chronic relapsing inflammatory optic neuritis | 1 |
| Myasthenia gravis | 1 |
| Neurosarcoidosis | 1 |
| Immune-mediated pachymeningitis | 1 |
| Multiple sclerosis | 1 |
| **Solid organ transplantation** | **n=20 (26%)** |
| Kidney transplantation | 13 |
| Liver transplantation | 3 |
| Heart transplantation | 3 |
| Hepatorenal transplantation | 1 |
| **Immune-hematologic-rheumatic diseases** | **n=24 (31%)** |
| Systemic lupus erythematosus | 8 |
| Autoimmune hemolytic anemia | 2 |
| Hemophagocytic syndrome | 2 |
| Juvenile dermatomyositis | 2 |
| Pemphigus | 2 |
| Di George syndrome | 1 |
| Combined immunodeficiency | 1 |
| Primary immunodeficiency and autoimmune hemolytic anemia | 1 |
| Juvenile idiopathic arthritis | 1 |
| Thrombocytopenia | 1 |
| ANCA-associated vasculitis | 1 |
| Juvenile systemic sclerosis | 1 |
| Unknown | 1 |
| **Oncological diseases and HSCT** | **n=14 (18%)** |
| HSCT | 5 |
| Burkitt lymphoma | 4 |
| B-cell acute lymphoblastic leukemia | 2 |
| EBV-positive lymphoma | 1 |
| Diffuse large B-cell lymphoma | 1 |
| Hodgkin lymphoma | 1 |

**Abbreviations**: ANCA: Antineutrophil cytoplasmic antibodies; EBV: Epstein-Barr virus; HSCT: Hematopoietic stem-cell transplantation; NHL: Non-Hodgkin lymphoma

Supplementary Table 2. Rituximab infusion regimen based on patient diagnosis and/or indication.

| **Indication** | **Dosage mg/m^2^** | **Frequency** |
| --- | --- | --- |
| **Neurologic diseases** | | |
| CNS inflammatory diseases^a^: Induction dosing | 375 | 4 weekly doses |
|  | 500 | 2 biweekly doses |
| CNS inflammatory diseases^a^: Maintenance redosing schedule (if necessary) | 500/750* | Single dose every 6 months from induction or when CD19 ≥ 1% |
| First-line treatment refractory disease^b^: Induction dosing | 375 | 4 weekly doses |
|  | 500 | 2 biweekly doses |
| First-line treatment refractory disease^b^: Maintenance redosing schedule (if necessary) | 375 | Single dose every 4-10 months |
| **Solid organ transplantation** | | |
| Acute rejection therapy | 375 | Single dose |
| PTLD | 375 | 1 to 4 weekly doses |
| **Hematologic diseases** | | |
| Autoimmune thrombocytopenia* | 375 | 4 weekly doses |
| ITP | 375 | 4 weekly doses |
| AIHA* | 375 | 4 weekly doses |
| Severe Hemophilia | 375 | Single dose |
| **Rheumatic diseases** | | |
| SLE, JDM, JIA, JSS, ANCA associated vasculitis | 750 | 2 biweekly doses |
| **HSCT** | | |
| HSCT Pre-transplant conditioning; Immunosuppressive induction therapy; Conditioning regimen | 375 | Single dose |
| Relapse prophylaxis; Acute GVHD prophylaxis | 375 | Single dose |
| Cytopenia secondary HSCT | 375 | 2 to 4 weekly doses |
| Haploidentical Transplant | 200 | Single dose |
| **Oncological diseases** | | |
| High-grade B-cell NHL | 375 | 4 weekly doses |
| NLPHL* | 375 | 4 weekly doses |
| Burkitt lymphoma | 375 | 4 weekly doses |
| Type B ALL | 375 | 4 weekly doses |
| Diffuse large B-cell lymphoma | 375 | 4 weekly doses |

* Patients refractory to first-line treatment

^a^: CNS inflammatory diseases (NMOSD, MS, Neurosarcoidosis, Optic neuritis, pachymeningitis, ADEM)

^b^: First-line treatment refractory diseases (OMS, seronegative anti-Musk MG, autoimmune encephalitis)

**Abbreviations**: CNS: central nervous system; HSCT: hematopoietic stem-cell transplantation; GVHD: Graft-versus-host disease; PTLD: Post-transplant lymphoproliferative disorder; NHL: Non-Hodgkin lymphoma; NLPHL: Nodular lymphocyte predominant Hodgkin lymphoma; ALL: Acute lymphocytic leukemia; ITP: Autoimmune thrombocytopenic purpura; AIHA: Autoimmune hemolytic anemia; NMOSD: Neuromyelitis optica spectrum disorder; MS: multiple sclerosis; ADEM: Acute disseminated encephalomyelitis; OMS: Opsoclonus-myoclonus syndrome; MG: Myasthenia gravis; SLE: Systemic lupus erythematosus; JDM: Juvenile dermatomyositis; JIA: Juvenile idiopathic arthritis; JSS: Juvenile systemic sclerosis.

Supplementary Table 3. Operational definition of adverse drug reactions induced by rituximab

| **Immune system disorders** | |
| --- | --- |
| Anaphylactic shock | A disorder characterized by an acute inflammatory reaction resulting from the release of histamine and histamine-like substances from mast cells, causing a hypersensitivity immune response. Clinically, it presents with breathing difficulty, dizziness, hypotension, cyanosis and loss of consciousness and may lead to death. The corresponding symptoms were recorded in order to report and analyze them. |
| Hypogammaglobulinemia | A disorder characterized by a decrease in total serum gammaglobulin levels or at least of one of the immunoglobulins (IgG, IgA, IgM, IgE) below the lower limit of the reference range according to the patient’s age and sex. |
| **Cutaneous disorders** | |
| Rash | Macular or papular eruption or erythema with or without associated symptoms. |
| **Cardiovascular disorders** | |
| Tachycardia | A disorder characterized by dysrhythmia with a heart rate of 30 beats per minute higher than the baseline value prior to starting rituximab infusion. |
| Hypertension | An average systolic blood pressure and/or diastolic blood pressure equal or greater than the 95th percentile for sex, age, and height of the patient on at least two occasions during or after rituximab infusion with values within the reference range prior to infusion initiation. |
| Hypotension | An average systolic blood pressure and/or diastolic blood pressure equal or lower than the 95th percentile for sex, age, and height of the patient on at least two occasions during or after rituximab infusion with values within the reference range prior to infusion initiation. |
| **Respiratory disorders** | |
| Tachypnea | An increase in respiratory rate of 10 breaths per minute or more compared to baseline prior to rituximab infusion. |
| Dyspnea | A disorder characterized by an uncomfortable sensation of difficulty breathing. |
| **Metabolism disorders** | |
| Hypophosphatemia | Serum phosphate concentration below the lower limit of the reference range according to patient’s age. |
| **Blood and lymphatic system disorders** | |
| Febrile neutropenia | A disorder characterized by an absolute neutrophil count <1000/mm3 and a single temperature of >38.3 ºC or a sustained temperature of >=38 ºC for more than one hour. |
| Lymphopenia | A decrease in absolute lymphocyte count below the lower limit of the reference range according to patient’s sex and age. |
| Leukopenia | A decrease in absolute leukocyte count below the lower limit of the reference range according to patient’s sex and age. |
| Late-onset neutropenia | Grade III–IV neutropenia, in which absolute neutrophil count is less than 1.0×10^3^/Liter, occurring 4 weeks after the last Rituximab administration. |

Supplementary Table 4. Concomitant medication received by the study population

| **Medication** | **Neu** | **SOT** | **IHR** | **O-HSCT** |
| --- | --- | --- | --- | --- |
| Steroids | Pred (9) | Pred (19) | Pred (19)  Dexa (2) | Pred (6)  Dexa (6) |
| Immunosuppressive drugs | MMF (1) | MMF (9)  FK (10)  Aza (4)  Csa (1)  Sirol (1) | MMF (2)  Aza (2)  Sirol (1)  Csa (1)  Mtx (1) | MMF (1)  FK (3)  Sirol (2)  Mtx (3) |
| Anticonvulsants* | Levet (4)  Top (1)  Clob (2)  Valpro (1) | Levet (1) | - | Levet (2)  Clob (1) |
| Chemotherapy | Cyclophos (2) | - | - | Doxo (3) Cyclophos(5)  Vincri (4) Iphospha(5)  Mesna(6) Etoposide(4)  Clofarabine(1)  Mercaptopu(1) Citarab(3) |

**Abbreviations:** Aza: azathioprine; Citarab: citarabine; Clob: clobazam; Csa: cyclosporine; Cyclophos: cyclophosphamide; Dexa: dexamethasone; Doxo: doxorubicin; FK: tacrolimus; HSCT: Hematopoietic stem-cell transplantation; IHR: Immune-hematologic-rheumatic; Iphospha: iphosphamide; Levet: levetiracetam; Mercaptopu: mercaptopurina; Mtx: methotrexate; MMF: mycophenolate mofetil; Neu: neurologic diseases; O: oncologic diseases; Pred: prednisone; Sirol: sirolimus; SOT: solid-organ transplantation; Top: topiramato; Valpro: valproate sodium; Vincri: vincristine.

***** Anticonvulsants include levetiracetam, Topiramato, Clobazam and Valproic Acid.

Supplementary Table 5. Actions taken after the development of IRRs according to symptomatology

| Clinical event/ symptoms | Actions |
| --- | --- |
| Anaphylaxis | Definitive discontinuation of rituximab, administration of adrenaline, hydrocortisone, diphenhydramine, and expansion with normal saline solution. |
| Cardiovascular/ Respiratory | Interruption of the infusion (n=8, 72.7%), administration of steroids and diphenhydramine (n=9, 81.8%), and/or reduction of infusion rate (n=9, 81.8%). Two cases of hypotension resolved without discontinuation or reduction of the infusion rate or medication; one of these cases required oxygen. Patients with arterial hypertension (n=3) did not require discontinuation or changes in the infusion rate, but two were medicated with antihypertensive drugs (enalapril in one case, amlodipine in the other). |
| Cutaneous | Interruption of the infusion (8/18, 44.4%), administration of medication (13/18, 72.2%) and/or reduction of infusion rate (11/18, 61.1%). |
| Gastrointestinal | One patient with nausea and abdominal pain did not require interruption of the infusion or medication, but the infusion rate was reduced. |
| General | Dipyrone was administered in patients with dizziness (n=1) and headache (n=1). Also in headache, the rate was reduced until the infusion was completed. |
